# Medical Comorbidities Associated With Outcomes in Patients With Traumatic Epidural Hematomas

**DOI:** 10.7759/cureus.15514

**Published:** 2021-06-08

**Authors:** James Brazdzionis, Tye Patchana, Paras Savla, Stacey Podkovik, Jonathan Browne, Ai Ohno, Taha M Taka, Arnav Modi, Margaret Rose Wacker, Vladimir Cortez, Dan E Miulli

**Affiliations:** 1 Neurosurgery, Riverside University Health System Medical Center, Moreno Valley, USA; 2 Neurosurgery, California University of Science and Medicine, Colton, USA; 3 Neurosurgery, University of California Riverside, Riverside, USA; 4 Neurosurgery, Arrowhead Regional Medical Center, Colton, USA; 5 Neurosurgery, Desert Regional Medical Center, Palm Springs, USA

**Keywords:** comorbid disease, epidural hematoma, traumatic brain injury, length of stay, comorbidity

## Abstract

Background

Traumatic brain injury (TBI) is a frequently encountered neurosurgical pathology with significant morbidity and mortality. One such subtype is the epidural hematoma. Literature regarding the effects of comorbidities in TBI and epidural hematomas is limited.

Methodology

This was a single-center retrospective review of 50 consecutive patients admitted to a level two trauma center with epidural hematomas. Patients were identified using an internal trauma database. Patients were included if they were 18 years of age with a diagnosed epidural hematoma. Outcome variables of Glasgow coma scale (GCS), length of stay in the intensive care unit (ICU) and hospital, and requirement of a neurosurgical procedure were analyzed. Identification of the presence of diagnosed comorbidities was performed including common comorbidities such as obesity, diabetes, hypertension, hyperlipidemia, drug use, tobacco use, cancer, psychiatric disease, and renal disease. Correlations were evaluated using two-sided bivariate analysis (p < 0.05).

Results

A total of 50 patients were included for analysis. Significant correlations with a p-value less of than 0.05 were noted in initial GCS and cancer (r = -0.357, p = 0.011), requirements of an intracranial procedure with a history of gastrointestinal disease (r = 0.377, p = 0.007), and younger age (r = -0.306, p = 0.031). Increased ICU length of stay was related to a history of cancer (r = 0.494, p < 0.001), a history of respiratory disease (r = 0.427, p = 0.002), and a history of psychiatric disease (r = 0.297, p = 0.036). Increased hospital length of stay was related to psychiatric disorders (r = 0.285, p = 0.045). Discharge GCS was negatively associated with a history of hypertension (r = -0.374, p = 0.008), tobacco use (r = -0.417, p = 0.003), drug use (r = -0.294, p = 0.037), and history of cancer (r = -0.303, p = 0.032).

Discussion and Conclusions

In our 50 consecutive patient subset, selected comorbidities demonstrated significant relationships with outcome measures of GCS, need for a procedure, and lengths of stay in the hospital and ICU. Obtaining comorbidity information when available from families can better allow the clinician to optimize treatment and educate loved ones about the potential effects of these comorbidities on the overall health of the patient. Understanding these correlations may allow for a better understanding of the systemic effects of the pathophysiology of injury in epidural hematomas.

## Introduction

Epidural hematomas are frequently encountered extra-axial hemorrhage within the subset of traumatic brain injuries (TBIs) and are commonly encountered in neurosurgical practice. TBI affects approximately 1.7 million individuals within the United States yearly and contributes to nearly one-third of all mortalities related to the injury [1,2]. Research has been ongoing to investigate factors that may predict outcomes in the TBI population to better understand the disease process and educate patients and families. Common factors investigated for predictive capacity include clinical data such as Glasgow coma scale (GCS), pupillary changes, blood pressure, and other physiologic parameters, along with common laboratory values obtained on arrival, including measures of coagulopathy and anemia [3]. Beyond the clinical and measurable factors, the patient’s associated comorbidities may also play a role in determining outcomes. With the incidence of chronic conditions within the U.S. adult population approaching 50%, it is critical to gain an understanding of the effect of these comorbidities on patient outcomes in neurological pathologies [4]. Specifically, the impact of preexisting comorbidities on patient outcomes in TBI has seldom been reported and has demonstrated inconsistencies regarding prognostication. There is limited data on epidural hematomas within the TBI population. Therefore, the majority of the literature has extrapolated the effects of other factors to this population.

Within TBI, pathophysiological injury derives from the primary insult or the secondary insult. The primary insult refers to the immediate damage caused by the head trauma itself (e.g., motor vehicle accidents, fall), whereas the secondary insult refers to the damage caused by hypoxia, electrolyte/nutrient imbalances, and pressure changes following the primary injury. Due to the association of secondary insult with systemic conditions, preexisting comorbidities such as diabetes and hypertension may exacerbate secondary insult damage and lead to poor patient outcomes.

Studies have identified that hyperglycemia is an independent risk factor for mortality in TBI [5]. Aside from leading research to determine the effect of posttraumatic hyperglycemia, this finding prompted research to assess the correlation between diabetes mellitus (DM) and poor TBI outcomes. These studies demonstrated that DM patients who are victims of TBI demonstrate a significant increase in patient mortality rates [5]. Additionally, studies on type-2 DM patients who previously suffered TBI reported the condition to be a significant predictor of poor long-term neuropsychological and functional outcomes [6,7].

Blood pressure effects on TBI outcomes have primarily revolved around studies demonstrating that acute hypotension is a strong predictor of negative patient outcomes [3]. The use of illicit drugs and alcohol has been assessed in association with TBI, and a previously published literature review reported that nearly two-thirds of TBI patients have a history of previous or current substance abuse, while approximately half are intoxicated at the time of injury [8]. It is important to note that drug and alcohol intoxication at the time of injury has mixed results within the literature in relation to mortality rates, but is associated with decreased functional outcomes [3,9-11]. Furthermore, history of substance abuse has been correlated with TBI outcomes, including mortality, long-term functional outcomes, and other complications [10].

In this study, we attempted to analyze the association of medical comorbidities within neurosurgical patients presenting with epidural hematomas to overall patient outcomes. The goal of this analysis was to help ascertain which combinations of medical comorbidities were associated with poorer outcomes or longer lengths of stay to better understand the pathological process.

## Materials and methods

This was a retrospective, institutional review board-approved study evaluating the last 50 patients admitted with a diagnosis of an epidural hematoma beginning in 2020 at a level two trauma center. Patients were recruited through a review of our center’s internal trauma database. Patients were included if they were over the age of 18 and were diagnosed with an epidural hematoma on admission secondary to trauma. Medical record review identified variables and demographic information including age and sex. Outcome measures of GCS on admission and discharge, intensive care unit (ICU) length of stay, total hospital length of stay, and whether a neurosurgical procedure was performed were evaluated. The presence of any of the following comorbidities on admission was assessed for their relationship with the outcome: obesity, diabetes, hyperlipidemia, congenital heart disease, congestive heart failure, coronary artery disease, hypertension, atrial fibrillation, tobacco use, drug use, hypothyroidism, migraine history, pulmonary disease, gastrointestinal disease, abdominal trauma, orthopedic injury, history of cancer, hematologic disease, renal disease, psychiatric disease, history of a reproductive disorder, burn injury, or history of developmental delay. Data analysis was performed using an open-source statistical analysis software, GNU PSPP. Correlations were evaluated using two-sided Pearson correlation coefficients with bivariate correlations. Significance levels were set at a p-value of <0.05.

## Results

A total of 50 patients were included in the analysis. The average age was 37.56 ± 17.36 years, 44 patients were male, and six were female. The average initial GCS was 12.52 with a standard deviation (SD) of 3.44, ranging from a GCS of 3 to 15. A total of 19 (38%) patients required neurosurgical procedures during their hospitalization. The average ICU length of stay was 5.46 ± 7.17 days, with an average hospital stay of 12.08 ± 20.22 days. The average GCS improved to 14.32 on discharge with an SD of 2.05, ranging from 3 to 15. This information is tabulated in Table 1.

**Table 1:** Demographics and descriptive characteristics of the studied cohort.

Characteristic	
Age (years)	37.56 ± 17.36
Sex	
Male	44
Female	6
Initial Glasgow coma scale	12.52 ± 3.44
Discharge Glasgow coma scale	14.32 ± 2.05
Neurosurgical procedure required	
Yes	19
No	31
Length of stay in intensive care unit (days)	5.46 ± 7.17
Length of stay in hospital (days)	12.08 ± 20.22

Bivariate correlations were used to analyze the outcome measures and studied comorbidities. Initial GCS was inversely correlated with cancer history (r = -0.357, p = 0.011). Patients were more likely to undergo an intracranial procedure if they had a history of gastrointestinal disease (r = 0.377, p = 0.007), and demonstrated an inverse correlation with age (r = -0.306, p = 0.031). Increased ICU length of stay was correlated with a history of cancer (r = 0.494, p < 0.001), a history of respiratory disease (r = 0.427, p = 0.002), and a history of psychiatric disease (r = 0.297, p = 0.036). Overall hospital length of stay was only significantly related with a history of psychiatric disorder (r = 0.285, p = 0.045). Discharge GCS was negatively associated with a history of hypertension (r = -0.374, p = 0.008), tobacco use (r = -0.417, p = 0.003), drug use (r = -0.294, p = 0.037), and a history of cancer (r = -0.303, p = 0.032). These significant correlations are listed in Table 2.

**Table 2:** Significant correlations in the studied cohort of patients with epidural hematomas.

Categories with significant correlation	Pearson correlation coefficient (r)	P-value
Initial Glasgow coma scale and history of cancer	-0.357	0.011
Underwent neurosurgical procedure and history of gastrointestinal disease	0.377	0.007
Underwent neurosurgical procedure and age	-0.306	0.031
Intensive care unit length of stay and history of cancer	0.494	<0.001
Intensive care unit length of stay and history of respiratory disease	0.427	0.002
Intensive care unit length of stay and history of psychiatric disease	0.297	0.036
Hospital length of stay and history of psychiatric disorder	0.285	0.045
Discharge Glasgow coma scale and history of hypertension	-0.374	0.008
Discharge Glasgow coma scale and tobacco use	-0.417	0.003
Discharge Glasgow coma scale and drug use	-0.294	0.037
Discharge Glasgow coma scale and history of cancer	-0.303	0.032

## Discussion

Outcomes in TBI have long been sought to be optimized through supportive care and reduction of secondary injury. In trauma patients, it can be difficult to obtain a comprehensive history related to their underlying comorbid conditions and medications, and in many cases, laboratory values and clinical examination are the treating surgeon’s only resources.

Our study found that patients with a history of malignancy had lower initial GCS, lower discharge GCS, and longer lengths of stay within the ICU (p < 0.05). This information as markers for severity of injury and outcome seemingly correlates with one of the more recent models in evaluating mortality in patients greater than 65 years old with TBI, which found that factors including history of pulmonary disease, malignancy, renal disease, or cardiac disease were related to increased mortality [12]. Our cohort, however, was on average younger (average age: 37.56 years) than the previously referenced trial.

We additionally found that patients with gastrointestinal diseases were more likely to require a neurosurgical procedure. Gastrointestinal processes that increase intraabdominal pressure have long been associated with increased intracranial pressure, especially as noted in severe disease processes such as abdominal compartment syndrome [13]. Although gastrointestinal processes were not stratified by specific disease in our cohort, the underlying principle of increased intra-abdominal pressure, either due to pain from the underlying disease or the disease itself, may biologically be related to increased intracranial pressure leading to additional neurosurgical procedures.

Our study found that younger patients were more likely to undergo a neurosurgical procedure than older patients diagnosed with an epidural hematoma. If undergoing a neurosurgical procedure is an adequate correlate to the severity of the injury, this coincides with the literature that has found higher degrees of injury in younger (aged 15-19 years) or much older patients (aged >75 years) with TBI than those in between [3].

Patients with any diagnosed respiratory disease had longer ICU lengths of stay; however, the overall length of stay was not affected. Pulmonary disease itself is a predictor of the need for mechanical ventilation in the standard ICU population, which may be an explanation for the correlation noted in our patient cohort [14]. Furthermore, in TBI, acute lung injury and neurogenic pulmonary edema is a well-documented phenomenon that leads to increased lengths of stay and morbidity [15]. With coexisting comorbid pulmonary disease, the patient’s compensatory mechanisms for acute-onset pathology may be limited, leading to increased ICU lengths of stay.

Psychiatric disease had a significant correlation with both ICU and overall hospital length of stays. Comorbid psychiatric disease has previously been studied in other common medical conditions such as chronic obstructive pulmonary disease and heart disease and has been found to be related to increased lengths of stay [16]. There are additional psychosocial factors at play in many patients with underlying psychiatric disease that may affect their length of stays that need to be considered. If the clinician obtains information earlier on during the admission, increased lengths of stay may theoretically be avoided with early case management utilization, ensuring that the patient obtains medications for their psychiatric disease and are appropriately given to avoid psychiatric complications.

Tobacco use was significantly correlated with decreased discharge GCS compared to the rest of the sample population. The recovery of smokers from TBI has previously been reported in the literature with mixed results demonstrating decreased functional outcomes in some studies but not finding significant differences in others [17-19]. It has been shown that tobacco use itself causes damage to the blood-brain barrier (BBB) [17]. These concomitant effects of injury from tobacco use and TBI result in a compound BBB injury that may explain the decreased recovery from TBI at discharge.

As described previously, substance use has been implicated in affecting outcomes from TBI [8,11]. Previous literature, however, has not developed a consensus on its overall effects. Our data found that substance use (not defined by type) was additionally associated with decreased GCS at discharge compared to the remaining cohort. This seems to imply that substance use may cause intrinsic injury to the neuronal cells, which do not appear to recover from their concomitant trauma as effectively compared to patients who do not have this associated comorbidity.

History of hypertension was correlated to a lower GCS at discharge compared to patients without hypertension. It is well known that hypotension is a significant cause of morbidity and mortality within the brain trauma realm [3,20]. In contrast, hypertension is more controversial due to the thought that arterial hypertension may promote the maintenance of cerebral perfusion pressure [20]. However, recent literature has shown that after TBI, hypertension, especially after the injury, is associated with poorer outcomes [12,20]. This has been theorized to be due to both the maintenance of cerebral perfusion pressure to overcome the increase in intracranial pressure (which is associated with a poorer outcome) as well as from a catecholamine surge [20]. The majority of these studies, however, investigated either inpatient blood pressure or prehospital blood pressure. Our study investigated preexisting hypertension as a comorbidity. It may be theorized that preexisting hypertension may not allow the body to appropriately compensate for the increased demand from the ischemic cerebrum from the TBI. This may result in impaired autoregulation which may theoretically lead to a poorer outcome and lower GCS at discharge.

Overall, for the treating physician, these comorbidities by themselves should not dictate care. However, the understanding that they may be associated with longer lengths of stay, poorer outcomes, or increased need for procedures may allow for a higher concern for related complications and a better understanding of outcomes. Furthermore, this understanding may allow the physician to better educate patients and families about their loved ones. Ultimately, primary prevention is the best course to reduce the potential for these comorbidities, and a multidisciplinary approach to promote high-quality primary care and risk factor reduction after the injury may help reduce these potential risk factors within the TBI population.

Limitations

This study was a retrospective review and therefore not all patients were interviewed at the time of data collection. Thus, some patients may not have reported a complete medical history. Furthermore, this was a subset sample of 50 patients, and therefore with a larger sample size, power may be increased to further look at the effect of these factors. Overall mortality was not investigated in this study, nor were long-term functional outcome measures. Future studies need to prospectively investigate these comorbidities regarding their effects on long-term functional outcomes. Furthermore, comorbidities may be related to diseases pathologically and may commonly present concomitantly. This interplay was not examined in our review. Radiographic features of each epidural hematoma were also not factored into our analysis which may play a role in patient outcomes.

## Conclusions

In our patient cohort, several comorbidities were found to be correlated with various outcome measures in patients with traumatic epidural hematomas. Younger age was significantly correlated to undergoing a neurosurgical procedure. Patients with gastrointestinal diseases were more likely to undergo a neurosurgical procedure, patients with respiratory diseases were significantly more likely to have longer ICU but not hospital lengths of stay, and patients with psychiatric disorders were significantly more likely to have longer ICU and hospital lengths of stay. Further histories of hypertension, tobacco use, and substance use were both significantly associated with a lower discharge GCS than the remaining cohort. Overall, patients with a history of malignancy had lower GCS on admission, lower GCS on discharge, and longer ICU lengths of stay. The treating traumatologist, neurosurgeon, and neurointensivist may consider using these comorbidities and potential effects on these differing outcome measures in discussions with families to allow them to better understand the effects of the entire body on the patient’s ability to recover from injury in the acute phase.
